# Phenyl bis­(*m*-tolyl­amido)­phosphinate

**DOI:** 10.1107/S1600536811024846

**Published:** 2011-07-02

**Authors:** Mehrdad Pourayoubi, Hossein Eshtiagh-Hosseini, Monireh Negari, Marek Nečas

**Affiliations:** a Department of Chemistry, Ferdowsi University of Mashhad, Mashhad 91779, Iran; b Department of Chemistry, Faculty of Science, Masaryk University, Kotlarska 2, Brno CZ-61137, Czech Republic

## Abstract

The P atom of the title compound, C_20_H_21_N_2_O_2_P, has a distorted tetra­hedral configuration; the bond angles at P are in the range 96.11 (6)–117.32 (8)°. The N atom exhibits *sp* ^2^ character. In the crystal, mol­ecules are connected *via* N—H⋯O hydrogen bonds into bands along the *a* axis, consisting of *R*_2_^2^(8) rings.

## Related literature

For background to compounds having a P(=O)(O)(N)(N) skeleton, see: Sabbaghi *et al.* (2010 ▶). For bond lengths in related structures, see: Ghadimi *et al.* (2009 ▶); Rudd *et al.* (1996 ▶). For graph-set notation, see Bernstein *et al.* (1995 ▶).
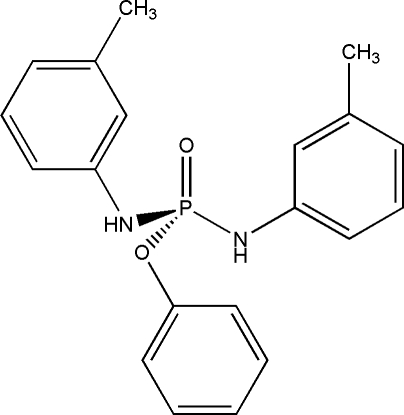


## Experimental

### 

#### Crystal data

C_20_H_21_N_2_O_2_P
*M* *_r_* = 352.36
Orthorhombic, 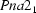

*a* = 10.1930 (2) Å
*b* = 16.8789 (3) Å
*c* = 10.4588 (3) Å
*V* = 1799.40 (7) Å^3^
*Z* = 4
Mo *K*α radiation
μ = 0.17 mm^−1^
*T* = 120 K
0.30 × 0.30 × 0.20 mm

#### Data collection

Oxford Diffraction Xcalibur Sapphire2 diffractometer
Absorption correction: multi-scan (*CrysAlis RED*; Oxford Diffraction, 2009 ▶) *T*_min_ = 0.959, *T*_max_ = 1.000
20798 measured reflections
2819 independent reflections
2647 reflections with *I* > 2σ(*I*)
*R*_int_ = 0.018

#### Refinement

*R*[*F* ^2^ > 2σ(*F* ^2^)] = 0.022
*wR*(*F* ^2^) = 0.062
*S* = 1.05
2819 reflections
228 parameters
1 restraint
H-atom parameters constrained
Δρ_max_ = 0.17 e Å^−3^
Δρ_min_ = −0.23 e Å^−3^
Absolute structure: Flack (1983 ▶), 1140 Friedel pairs
Flack parameter: −0.04 (7)

### 

Data collection: *CrysAlis CCD* (Oxford Diffraction, 2009 ▶); cell refinement: *CrysAlis RED* (Oxford Diffraction, 2009 ▶); data reduction: *CrysAlis RED*; program(s) used to solve structure: *SHELXS97* (Sheldrick, 2008 ▶); program(s) used to refine structure: *SHELXL97* (Sheldrick, 2008 ▶); molecular graphics: *Mercury* (Macrae *et al.*, 2008 ▶); software used to prepare material for publication: *enCIFer* (Allen *et al.*, 2004 ▶).

## Supplementary Material

Crystal structure: contains datablock(s) I, global. DOI: 10.1107/S1600536811024846/ld2017sup1.cif

Structure factors: contains datablock(s) I. DOI: 10.1107/S1600536811024846/ld2017Isup2.hkl

Additional supplementary materials:  crystallographic information; 3D view; checkCIF report

## Figures and Tables

**Table 1:** Hydrogen-bond geometry (Å, °)

*D*—H⋯*A*	*D*—H	H⋯*A*	*D*⋯*A*	*D*—H⋯*A*
N1—H1*B*⋯O1^i^	0.88	2.14	2.9917 (16)	162
N2—H2*A*⋯O2^ii^	0.88	2.53	3.3929 (16)	166

Symmetry codes: (i) 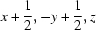; (ii) 
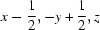
.

**Fig. 1.**
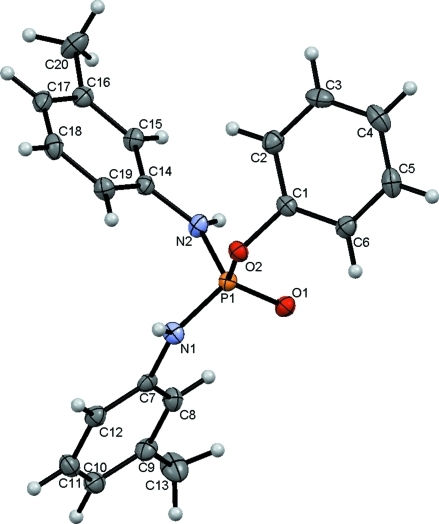
The molecular structure of the title compound with ellipsoids shown at the 50% probability level.

**Fig. 2.**
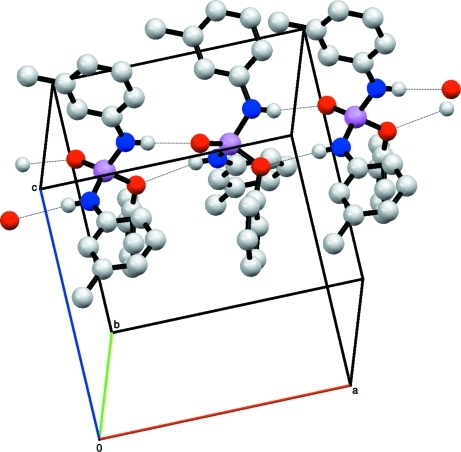
Part of the crystal structure with hydrogen bonds shown as dotted lines (the C—H hydrogen atoms are omitted for clarity).

## supplementary crystallographic information

### Comment

Structure determination of the title compound, P(O)[OC_6_H_5_][NHC_6_H_4_(*m*-CH_3_)]_2_ (Fig. 1), was performed as a part of a project in our laboratory on the synthesis of compounds having a P(═O)(O)(N)(N) skeleton (Sabbaghi *et al.*, 2010). Single crystals of title compound were obtained from a mixture of CHCl_3_/CH_3_OH (4:1 *v*/*v*) after slow evaporation at room temperature.

The P═O (1.4692 (11) Å), P—O (1.5941 (12) Å) and P—N (1.6374 (15) Å & 1.6469 (13) Å) bond lengths are usual for this category of compounds (Ghadimi *et al.*, 2009). The P atom has a distorted tetrahedral configuration (Fig. 1), as it has been noted for the other phosphoramidates and their chalco-derivatives (Rudd *et al.*, 1996). The bond angles at the P atom vary in the range from 96.11 (6)°-117.32 (8)°. The C1—O2—P1, C7—N1—P1 and C14—N2—P1 angles are 120.49 (9)°, 127.29 (11)° and 130.85 (11)°, respectively.

Prior to the constrained refinement of the imino H atoms, we have tested a free refinement of these atoms localized from a difference Fourier map. The results suggested an almost perfect in-plane arrangement of both H atoms with the corresponding P,*N*,*C* atoms. Therefore, the imino groups were assigned a planar geometry to reduce the number of parameters and to maintain more appropriate N—H bond lengths.

One of the two N—H units adopts a *syn* orientation with respect to the phosphoryl group, while the other exists in an *anti* orientation.

In the crystal, each molecule is hydrogen-bonded to two adjacent molecules through N—H···O hydrogen bonds, building *R*_2_^2^(8) rings (Bernstein *et al.*, 1995), and forming linear arrangements parallel to [100] (Fig. 2, Table 1).

### Experimental

To a solution of phenyl dichlorophosphate (2.35 mmol) in chloroform (15 ml), a solution of *meta*-toluidine (9.40 mmol) in chloroform (30 ml) was added at 273 K. After 4 h of stirring, the solvent was evaporated in vacuum. The solid was washed with distilled water. Single crystals, suitable for crystallography, were obtained from a solution of the title compound in chloroform and methanol (4:1) after slow evaporation at room temperature. IR (KBr, cm^-1^): 3402, 3124, 2890, 2607, 1586, 1494, 1401, 1293, 1182, 1023, 946, 757.

### Refinement

Hydrogen atoms in phenyl and NH groups were positioned geometrically and refined as riding with their *U*_iso_ set to 1.2*U*_eq_ of the parent atoms. Hydrogen atoms in methyl groups were positioned geometrically and refined as rotating with their *U*_iso_ set to 1.5*U*_eq_ of the parent atoms.

### Crystal data

**Table:**

C_20_H_21_N_2_O_2_P	*D*_x_ = 1.301 Mg m^−^^3^
*M**_r_* = 352.36	Mo *K*α radiation, λ = 0.71073 Å
Orthorhombic, *P**n**a*2_1_	Cell parameters from 13732 reflections
*a* = 10.1930 (2) Å	θ = 3.0–27.2°
*b* = 16.8789 (3) Å	µ = 0.17 mm^−^^1^
*c* = 10.4588 (3) Å	*T* = 120 K
*V* = 1799.40 (7) Å^3^	BLOCK, colourless
*Z* = 4	0.30 × 0.30 × 0.20 mm
*F*(000) = 744	

### Data collection

**Table:**

Oxford Diffraction Xcalibur Sapphire2 diffractometer	2819 independent reflections
Radiation source: Enhance (Mo) X-ray Source	2647 reflections with *I* > 2σ(*I*)
graphite	*R*_int_ = 0.018
Detector resolution: 8.4353 pixels mm^-1^	θ_max_ = 25.0°, θ_min_ = 3.0°
ω scans	*h* = −12→12
Absorption correction: multi-scan (*CrysAlis RED*; Oxford Diffraction, 2009)	*k* = −20→20
*T*_min_ = 0.959, *T*_max_ = 1.000	*l* = −8→12
20798 measured reflections	

### Refinement

**Table:**

Refinement on *F*^2^	Secondary atom site location: difference Fourier map
Least-squares matrix: full	Hydrogen site location: inferred from neighbouring sites
*R*[*F*^2^ > 2σ(*F*^2^)] = 0.022	H-atom parameters constrained
*wR*(*F*^2^) = 0.062	*w* = 1/[σ^2^(*F*_o_^2^) + (0.046*P*)^2^] where *P* = (*F*_o_^2^ + 2*F*_c_^2^)/3
*S* = 1.05	(Δ/σ)_max_ = 0.001
2819 reflections	Δρ_max_ = 0.17 e Å^−^^3^
228 parameters	Δρ_min_ = −0.23 e Å^−^^3^
1 restraint	Absolute structure: Flack (1983), 1140 Friedel pairs
Primary atom site location: structure-invariant direct methods	Flack parameter: −0.04 (7)

### Special details

**Table:**

Geometry. All e.s.d.'s (except the e.s.d. in the dihedral angle between two l.s. planes) are estimated using the full covariance matrix. The cell e.s.d.'s are taken into account individually in the estimation of e.s.d.'s in distances, angles and torsion angles; correlations between e.s.d.'s in cell parameters are only used when they are defined by crystal symmetry. An approximate (isotropic) treatment of cell e.s.d.'s is used for estimating e.s.d.'s involving l.s. planes.
Refinement. Refinement of *F*^2^ against ALL reflections. The weighted *R*-factor *wR* and goodness of fit *S* are based on *F*^2^, conventional *R*-factors *R* are based on *F*, with *F* set to zero for negative *F*^2^. The threshold expression of *F*^2^ > σ(*F*^2^) is used only for calculating *R*-factors(gt) *etc*. and is not relevant to the choice of reflections for refinement. *R*-factors based on *F*^2^ are statistically about twice as large as those based on *F*, and *R*- factors based on ALL data will be even larger.

### Fractional atomic coordinates and isotropic or equivalent isotropic displacement parameters (Å^2^)

**Table:**

	*x*	*y*	*z*	*U*_iso_*/*U*_eq_	
P1	0.72349 (4)	0.24971 (2)	0.91839 (5)	0.01542 (11)	
O1	0.61830 (10)	0.19795 (6)	0.96582 (11)	0.0194 (3)	
O2	0.82530 (10)	0.20973 (6)	0.82235 (12)	0.0184 (3)	
N1	0.83286 (12)	0.28002 (7)	1.02210 (16)	0.0179 (3)	
H1B	0.9156	0.2753	0.9993	0.022*	
N2	0.65260 (12)	0.32433 (7)	0.84411 (15)	0.0193 (3)	
H2A	0.5663	0.3236	0.8462	0.023*	
C1	0.78034 (13)	0.16622 (9)	0.71528 (18)	0.0171 (4)	
C2	0.76338 (16)	0.20366 (10)	0.60039 (19)	0.0240 (4)	
H2B	0.7740	0.2594	0.5937	0.029*	
C3	0.73032 (17)	0.15880 (11)	0.49380 (19)	0.0269 (4)	
H3A	0.7164	0.1839	0.4136	0.032*	
C4	0.71767 (15)	0.07733 (11)	0.5049 (2)	0.0268 (4)	
H4A	0.6978	0.0464	0.4315	0.032*	
C5	0.73373 (15)	0.04090 (10)	0.6218 (2)	0.0250 (4)	
H5A	0.7240	−0.0149	0.6287	0.030*	
C6	0.76376 (14)	0.08496 (10)	0.72845 (18)	0.0210 (4)	
H6A	0.7730	0.0603	0.8096	0.025*	
C7	0.80937 (15)	0.31292 (8)	1.14394 (17)	0.0167 (4)	
C8	0.68462 (15)	0.31324 (9)	1.19924 (19)	0.0217 (4)	
H8A	0.6124	0.2915	1.1538	0.026*	
C9	0.66553 (16)	0.34526 (9)	1.32059 (18)	0.0230 (4)	
C10	0.77167 (16)	0.37699 (9)	1.38620 (19)	0.0243 (4)	
H10A	0.7595	0.3989	1.4690	0.029*	
C11	0.89519 (16)	0.37666 (9)	1.33103 (18)	0.0240 (4)	
H11A	0.9674	0.3985	1.3764	0.029*	
C12	0.91464 (15)	0.34502 (9)	1.21091 (18)	0.0208 (4)	
H12A	0.9998	0.3451	1.1740	0.025*	
C13	0.53035 (17)	0.34519 (12)	1.3794 (2)	0.0357 (5)	
H13A	0.5369	0.3315	1.4703	0.054*	
H13B	0.4752	0.3061	1.3356	0.054*	
H13C	0.4911	0.3979	1.3706	0.054*	
C14	0.70709 (15)	0.38945 (9)	0.77755 (18)	0.0177 (4)	
C15	0.62148 (15)	0.44120 (9)	0.71729 (17)	0.0198 (4)	
H15A	0.5298	0.4319	0.7233	0.024*	
C16	0.66624 (16)	0.50657 (9)	0.64807 (17)	0.0212 (4)	
C17	0.80128 (15)	0.51933 (9)	0.64137 (18)	0.0219 (4)	
H17A	0.8346	0.5635	0.5953	0.026*	
C18	0.88614 (15)	0.46829 (9)	0.70120 (18)	0.0232 (4)	
H18A	0.9778	0.4781	0.6959	0.028*	
C19	0.84227 (15)	0.40269 (9)	0.76930 (18)	0.0201 (4)	
H19A	0.9027	0.3677	0.8093	0.024*	
C20	0.57089 (18)	0.55834 (10)	0.57636 (19)	0.0327 (5)	
H20A	0.6048	0.6126	0.5728	0.049*	
H20B	0.4860	0.5581	0.6204	0.049*	
H20C	0.5598	0.5380	0.4893	0.049*	

### Atomic displacement parameters (Å^2^)

**Table:**

	*U*^11^	*U*^22^	*U*^33^	*U*^12^	*U*^13^	*U*^23^
P1	0.01458 (18)	0.01538 (17)	0.0163 (2)	0.00013 (14)	0.0010 (2)	−0.00022 (17)
O1	0.0181 (5)	0.0198 (5)	0.0203 (7)	−0.0003 (4)	0.0018 (5)	0.0011 (5)
O2	0.0169 (5)	0.0202 (5)	0.0180 (7)	−0.0005 (4)	0.0004 (5)	−0.0025 (5)
N1	0.0127 (6)	0.0217 (6)	0.0195 (8)	0.0011 (5)	0.0024 (6)	−0.0030 (6)
N2	0.0130 (6)	0.0196 (6)	0.0253 (9)	−0.0001 (5)	0.0007 (6)	0.0031 (6)
C1	0.0131 (7)	0.0208 (7)	0.0175 (10)	0.0007 (6)	0.0014 (7)	−0.0034 (7)
C2	0.0274 (9)	0.0203 (8)	0.0242 (12)	0.0009 (6)	0.0020 (8)	−0.0008 (8)
C3	0.0280 (9)	0.0370 (10)	0.0158 (11)	0.0028 (8)	0.0005 (8)	0.0005 (9)
C4	0.0189 (8)	0.0329 (9)	0.0287 (12)	−0.0002 (7)	−0.0021 (8)	−0.0120 (9)
C5	0.0182 (8)	0.0197 (8)	0.0372 (13)	−0.0017 (6)	0.0017 (8)	−0.0078 (9)
C6	0.0182 (8)	0.0218 (8)	0.0231 (11)	−0.0003 (6)	0.0002 (7)	0.0024 (8)
C7	0.0198 (7)	0.0129 (7)	0.0174 (10)	0.0019 (6)	0.0017 (7)	0.0026 (7)
C8	0.0191 (8)	0.0210 (7)	0.0250 (11)	−0.0026 (6)	0.0013 (7)	−0.0013 (8)
C9	0.0257 (9)	0.0214 (8)	0.0220 (10)	−0.0005 (6)	0.0066 (8)	−0.0007 (8)
C10	0.0333 (9)	0.0197 (7)	0.0197 (12)	0.0007 (7)	0.0019 (8)	−0.0024 (7)
C11	0.0246 (8)	0.0235 (8)	0.0240 (11)	−0.0019 (6)	−0.0042 (8)	−0.0030 (8)
C12	0.0190 (8)	0.0212 (7)	0.0222 (10)	−0.0005 (6)	0.0009 (7)	0.0018 (7)
C13	0.0302 (10)	0.0449 (10)	0.0322 (13)	−0.0055 (8)	0.0109 (9)	−0.0121 (9)
C14	0.0204 (8)	0.0161 (7)	0.0167 (10)	−0.0013 (6)	0.0022 (7)	−0.0024 (7)
C15	0.0159 (7)	0.0207 (7)	0.0229 (10)	0.0001 (6)	0.0026 (7)	−0.0005 (8)
C16	0.0254 (9)	0.0190 (7)	0.0190 (10)	0.0015 (6)	0.0038 (7)	−0.0015 (8)
C17	0.0269 (9)	0.0189 (8)	0.0198 (11)	−0.0057 (7)	0.0048 (8)	−0.0004 (8)
C18	0.0182 (8)	0.0232 (8)	0.0281 (12)	−0.0050 (6)	0.0036 (8)	−0.0038 (8)
C19	0.0164 (8)	0.0201 (7)	0.0240 (10)	0.0015 (6)	−0.0009 (7)	−0.0014 (7)
C20	0.0334 (10)	0.0279 (9)	0.0368 (13)	0.0033 (7)	0.0044 (9)	0.0118 (9)

### Geometric parameters (Å, °)

**Table:**

P1—O1	1.4692 (11)	C9—C10	1.389 (2)
P1—O2	1.5941 (12)	C9—C13	1.509 (2)
P1—N1	1.6374 (15)	C10—C11	1.385 (2)
P1—N2	1.6469 (13)	C10—H10A	0.9500
O2—C1	1.416 (2)	C11—C12	1.379 (3)
N1—C7	1.410 (2)	C11—H11A	0.9500
N1—H1B	0.8800	C12—H12A	0.9500
N2—C14	1.415 (2)	C13—H13A	0.9800
N2—H2A	0.8800	C13—H13B	0.9800
C1—C2	1.369 (3)	C13—H13C	0.9800
C1—C6	1.389 (2)	C14—C15	1.386 (2)
C2—C3	1.389 (3)	C14—C19	1.399 (2)
C2—H2B	0.9500	C15—C16	1.396 (2)
C3—C4	1.386 (3)	C15—H15A	0.9500
C3—H3A	0.9500	C16—C17	1.395 (2)
C4—C5	1.378 (3)	C16—C20	1.507 (2)
C4—H4A	0.9500	C17—C18	1.372 (2)
C5—C6	1.375 (3)	C17—H17A	0.9500
C5—H5A	0.9500	C18—C19	1.390 (2)
C6—H6A	0.9500	C18—H18A	0.9500
C7—C12	1.391 (2)	C19—H19A	0.9500
C7—C8	1.397 (2)	C20—H20A	0.9800
C8—C9	1.393 (3)	C20—H20B	0.9800
C8—H8A	0.9500	C20—H20C	0.9800
			
O1—P1—O2	115.86 (6)	C11—C10—C9	120.06 (17)
O1—P1—N1	117.32 (8)	C11—C10—H10A	120.0
O2—P1—N1	96.11 (6)	C9—C10—H10A	120.0
O1—P1—N2	107.09 (6)	C12—C11—C10	120.77 (16)
O2—P1—N2	108.19 (7)	C12—C11—H11A	119.6
N1—P1—N2	111.85 (7)	C10—C11—H11A	119.6
C1—O2—P1	120.49 (9)	C11—C12—C7	119.90 (15)
C7—N1—P1	127.29 (11)	C11—C12—H12A	120.0
C7—N1—H1B	116.4	C7—C12—H12A	120.0
P1—N1—H1B	116.4	C9—C13—H13A	109.5
C14—N2—P1	130.85 (11)	C9—C13—H13B	109.5
C14—N2—H2A	114.6	H13A—C13—H13B	109.5
P1—N2—H2A	114.6	C9—C13—H13C	109.5
C2—C1—C6	121.86 (17)	H13A—C13—H13C	109.5
C2—C1—O2	119.73 (13)	H13B—C13—H13C	109.5
C6—C1—O2	118.25 (16)	C15—C14—C19	119.44 (15)
C1—C2—C3	118.92 (15)	C15—C14—N2	117.78 (13)
C1—C2—H2B	120.5	C19—C14—N2	122.77 (15)
C3—C2—H2B	120.5	C14—C15—C16	121.87 (14)
C4—C3—C2	119.75 (18)	C14—C15—H15A	119.1
C4—C3—H3A	120.1	C16—C15—H15A	119.1
C2—C3—H3A	120.1	C17—C16—C15	118.07 (15)
C5—C4—C3	120.38 (18)	C17—C16—C20	121.46 (15)
C5—C4—H4A	119.8	C15—C16—C20	120.37 (14)
C3—C4—H4A	119.8	C18—C17—C16	120.14 (15)
C6—C5—C4	120.32 (15)	C18—C17—H17A	119.9
C6—C5—H5A	119.8	C16—C17—H17A	119.9
C4—C5—H5A	119.8	C17—C18—C19	122.08 (14)
C5—C6—C1	118.72 (17)	C17—C18—H18A	119.0
C5—C6—H6A	120.6	C19—C18—H18A	119.0
C1—C6—H6A	120.6	C18—C19—C14	118.40 (15)
C12—C7—C8	119.48 (16)	C18—C19—H19A	120.8
C12—C7—N1	118.51 (14)	C14—C19—H19A	120.8
C8—C7—N1	122.01 (15)	C16—C20—H20A	109.5
C9—C8—C7	120.39 (16)	C16—C20—H20B	109.5
C9—C8—H8A	119.8	H20A—C20—H20B	109.5
C7—C8—H8A	119.8	C16—C20—H20C	109.5
C10—C9—C8	119.41 (15)	H20A—C20—H20C	109.5
C10—C9—C13	120.67 (17)	H20B—C20—H20C	109.5
C8—C9—C13	119.92 (16)		

### Hydrogen-bond geometry (Å, °)

**Table:**

*D*—H···*A*	*D*—H	H···*A*	*D*···*A*	*D*—H···*A*
N1—H1B···O1^i^	0.88	2.14	2.9917 (16)	162
N2—H2A···O2^ii^	0.88	2.53	3.3929 (16)	166

Symmetry codes: (i) *x*+1/2, −*y*+1/2, *z*; (ii) *x*−1/2, −*y*+1/2, *z*.
